# Knowledge, attitudes, and practices of oral cancer prevention among dental students and interns: an online cross‑sectional questionnaire in Palestine

**DOI:** 10.1186/s12903-022-02415-8

**Published:** 2022-09-05

**Authors:** Rola Muhammed Shadid, Mohammad Amid Abu Ali, Omar Kujan

**Affiliations:** 1 Department of Prosthodontics, College of Dentistry, Arab American University, P.O. Box: 240, Jenin, Palestinian Territory Palestinian Territory; 2 Private practice, Palestinian Territory, Palestinian Territory; 3 Department of Oral and Maxillofacial surgery, College of Dentistry, Arab American University, Jenin, Palestinian Territory; 4 Lead Discipline in Oral Pathology, UWA Dental School, University of Western Australia, Nedlands, WA Australia

**Keywords:** Oral cancer, Knowledge, Practice, Attitude, Dental students, Interns, Palestine

## Abstract

**Background:**

Oral cancer is frequently characterized with an aggressive behavior and an unfavorable prognosis; however, it is generally associated with promising prognosis if detected early. Therefore, this study aimed to assess knowledge, practices, and attitudes toward oral cancer prevention among dental students and interns; and to investigate the factors that influence their practices of oral cancer screening or prevention.

**Material and methods:**

A cross-sectional questionnaire-based survey was conducted between March and April of 2022 on the fourth- and fifth-year undergraduate dental students and interns in the College of Dentistry at Arab American University in Palestine. A 48-item questionnaire which has 4 sections: demographics, knowledge, practices, and attitudes toward oral cancer prevention and early detection was sent to all eligible participants (N = 570).

**Results:**

The response rate was 68.7% (N = 351). About 66.8% of the respondents had poor knowledge about oral cancer and its risk factors, and 85.5% had a poor practice of oral cancer early detection and prevention; however, the majority of the respondents (81.1%) had shown favorable attitudes toward oral cancer prevention. Interns had significantly better knowledge and attitude scores compared to the undergraduate dental students (*P* < 0.05). Lack of training, time, confidence, and effectiveness were stated among the barriers to oral cancer screening.

**Conclusions:**

Most of the participants surveyed in this study appeared to lack adequate knowledge and skills in oral cancer prevention and early detection; however, they seemed to have good motivation and a good attitude toward oral cancer prevention training.

## Background

Lip and oral cavity cancers are considered a main global health problem representing the 16th most common neoplasm globally, with almost 377,713 new cases and about 177,757 deaths registered in 2020 [1]. However, most of the new cases of oral cancers are reported in the developing countries [2]. Kujan et al. based on GLOBOCAN 2012 projection, estimated that the number of new cases and the mortality rate of oral cancer will duplicate in the Middle East and North Africa (MENA) region, where most countries in this region are from the developing world, by the year 2030. This contrasts with the incidence and mortality rate of the oral cancer globally where it is estimated to increase by only 50% in the same time span [3].

In Palestine as part of the MENA region, the newly reported cases of oral cavity and oropharyngeal cancer were estimated at around 90 and the associated deaths were 35 in 2012 [4].

Squamous cell carcinoma has been found to be the most common type of the lip and oral cavity cancers representing about 90% [5], and it is regarded to be a multifactorial disease [6, 7]. Heavy tobacco smoking, alcohol drinking, viral infection by human papilloma virus (HPV), and genetic instability are considered to be the main risk factors in the MENA region [7–11]. Dietary, occupational, and environmental risk factors might also contribute to oral cancer development [12]. However, tobacco smoking that is an endemic habit in the MENA region is considered the most significant risk factor. For example, the prevalence of tobacco smoking among the Palestinian male population is about 40% [12].

Oral cancers are most prevalent on the lower lip, lateral border of the tongue, and floor of the mouth. They affect mainly the middle-aged and elderly men with the highest occurrence in the sixth to eighth decades of life [3]. Although oral cancers are frequently characterized with an aggressive behavior and unfavorable prognosis [13, 14], they are generally associated with promising prognosis if detected early [15]. These lesions can be prevented by either lessening exposure to risk factors or detection and surveillance of oral potentially malignant disorders [16]. Unfortunately, a recent systematic review demonstrated that oral cancers are commonly ignored by healthcare providers and the resulting delay in referrals is a major contributor to disease’s advanced stages [17]. General dental practitioners play a vital role in the prevention and early detection of oral cancer due to their forefront contact with patients [18].

Several studies worldwide investigated oral cancer awareness, knowledge, practices, and attitudes among qualified practicing and future dentists [18–32]. Some of these studies revealed poor or inadequate level of knowledge, attitudes, or practices of oral cancer prevention [22, 30–32], implying the need for improving the oral cancer curricula in undergraduate and graduate dental courses and for conducting more continuing education programs for healthcare professionals to improve the knowledge and attitudes toward oral cancer prevention and early detection.

Since the incidence of oral cancer is increasing in the MENA region [3] due to incremental trends of highly associated risk factors such as smoking and alcohol consumption [12], and owing to the vital role played by the general dental practitioners in the prevention and detection of oral cancer lesions at early stages, and because no study till now was conducted in Palestine to assess the dentist’s oral cancer awareness, this cross-sectional study aimed to assess the knowledge, practices, and attitudes toward oral cancer prevention among students and interns in the College of Dentistry at the Arab American University (AAUP); and to investigate the factors that influence their practices of oral cancer prevention or early detection.

## Methods

This cross-sectional questionnaire-based study was conducted and reported in accordance with CHERRIES guidelines [33] between March and April of 2022, and targeted the fourth- and fifth-year undergraduate dental students and interns in the College of Dentistry at AAUP. A total of 511 undergraduate students and 59 interns were deemed as eligible participants. The study was approved by the Institutional Review Board (IRB), College of Dentistry, Arab American University (2022/A/3/N), and was conducted in agreement with the Declaration of Helsinki guidelines. All participants provided informed consent. The 48- item questionnaire used in the current study was established after reviewing previous studies [18, 23, 29], and it was pilot tested on a group of 20 undergraduate students and interns to verify its clarity and simplicity. The questionnaire content validity was evaluated by an expert in Oral Medicine and it was pre-validated in previous research work [18]. Reliability was assessed using the test–retest method in which 20 students and interns completed the questionnaire twice within two weeks. Outcomes of the two times were compared using Pearson’s correlation coefficient that has shown a significant stability coefficient suggesting a good test–retest reliability.

Regarding internal consistency between items in the survey, it was measured using the coefficient alpha "Cronbach’s alpha”. A Cronbach α = 0.785 was attained, entailing acceptable internal consistency.

The questionnaire was created online using Google Drive and a link was emailed and shared with all eligible participants in closed groups on social media. A cover letter accompanying the questionnaire explained the study aims, the methods of the study, and assured that the participation was voluntary, anonymous, and all information given would be confidential and used for research purposes only.

The questionnaire consisted of 47 close-ended questions and one open-ended question which were categorized into 4 sections: demographics, knowledge, practices, and attitudes toward oral cancer prevention and early detection. The first section included information about the demographics such as age, sex, and education level. The second section included questions about participants’ knowledge regarding oral cancer signs, symptoms, and risk factors (22 questions). A score of “1” was given for each correct answer on the questions, so the overall knowledge score ranged from 0–22. The current study used (13.2 /22) 60% as cut-off points [32], > 13.2 points was considered to have good knowledge and ≤ 13.2 points was considered to have poor knowledge of oral cancer.

The third part of the questionnaire included 12 questions about practices of oral cancer prevention and detection and one question about the claimed barriers for regular oral cancer screening. The best practice score was 12 while 7.2 represented a cut-off score; > 7.2 was regarded as good practice and ≤ 7.2 as poor practice.

In the final part of the questionnaire, participants were questioned about their attitudes and opinions about oral cancer prevention by responding to 10 statements using “strongly agree”, “agree”, “disagree”, and “strongly disagree”. A score of 1 designated positive attitude, while a score of 0 designated negative attitude, with 10 represented the maximum score. Six was set as cut-off score; > 6 was considered as a favorable attitude and ≤ 6 as unfavorable.

### Statistical analysis

Responses were assembled using the Google Drive Excel document, and data were analyzed statistically using the Statistical Package for Social Sciences (SPSS), version 22.0. Descriptive statistics of the mean, standard deviation and percentages were calculated for all continuous variables. The level of statistical significance was considered at *P* < 0.05.

The influence of sex and age on the knowledge, practice, and attitude scores was evaluated using independent samples t-test, and the influence of education level on the abovementioned variables was tested using One Way Analysis of Variance (ANOVA).

## Results

Of the 511 undergraduate dental students who were requested to participate, 351 completed the survey with a response rate of 68.7%; and 41 interns from 59 filled the survey with a 69.5% response rate. This represents an overall response rate of 68.8%. Most of the respondents were females (64.3%), ≤ 30 years old (94.1%), and undergraduate dental students (98.5%; Table 1).

**Table 1:** Characteristics of survey’s respondents (n = 392)

Variable	N	%
Age		
≤ 30	369	94.1
> 30	23	5.9
Gender		
Male	140	35.7
Female	252	64.3
Level of education		
4th-year	168	42.8
5th-year	183	46.7
Intern	41	10.5

Table 2 shows the number and percentage of undergraduate dental students and interns who correctly answered the knowledge questions. Table 3 shows the number and percentage of undergraduate dental students and interns who recognized high-risk factors for oral cancer. Table 4 reveals the number and percentage of undergraduate dental students and interns who claimed good practice and experience with oral cancer screening and referral at College of Dentistry. Table 5 demonstrates the number and percentage of undergraduate dental students and interns who exhibited positive attitudes toward oral cancer prevention.

**Table 2:** Number and percentage of undergraduate dental students and interns who correctly answered the knowledge questions

Question	Survey results						
	Male, n (%)			Female, n (%)			Overall, n (%)
	Fourth	Fifth	Intern	Fourth	Fifth	Intern	
What is the most common oral cancer?	47 (12.0)	47 (12.0)	18 (4.6)	74 (18.9)	117 (29.8)	18 (4.6)	321 (81.9)
Excluding the lip, which of the following are the two most common sites of oral cancer?	26 (6.6)	19 (4.9)	9 (2.3)	34 (8.7)	57 (14.5)	11 (2.8)	156 (39.8)
The most common age of the patients to develop oral cancer is:	20 (5.1)	21 (5.3)	13 (3.3)	41 (10.5)	58 (14.8)	10 (2.6)	163 (41.6)
The symptom most commonly experienced by a patient with an early oral cancer is:	15 (3.8)	26 (6.6)	6 (1.5)	38 (9.7)	50 (12.8)	11 (2.8)	146 (37.2)
Oral cancer lesions are most often diagnosed in which stage:	35 (8.9)	29 (7.4)	9 (2.3)	44 (11.2)	59 (15.1)	7 (1.8)	183 (46.7)
A lymph node most characteristic of oral cancer metastasis, when palpated, is:	34 (8.7)	27 (6.9)	7 (1.8)	62 (15.8)	80 (20.4)	15 (3.8)	225 (57.4)
Early oral cancer lesions usually appear as a:	35 (8.9)	36 (9.2)	19 (4.9)	69 (17.6)	91 (23.2)	17 (4.3)	267 (68.1)
Which area of the tongue is most likely to develop oral cancer?	10 (2.5)	25 (6.4)	10 (2.6)	15 (3.8)	47 (12.0)	9 (2.3)	116 (29.6)
When examining the tongue for oral cancer, the clinician should:	17 (4.3)	28 (7.1)	12 (3.1)	48 (12.3)	80 (20.4)	14 (3.6)	199 (50.8)
The mortality rate of oral cancer is greater among:	33 (8.4)	29 (7.4)	17 (4.4)	52 (13.3)	80 (20.4)	15 (3.8)	226 (57.7)

**Table 3:** Number and percentage of undergraduate dental students and interns who recognized hig-risk factors for oral cancer

Risk factor	Survey results						
	Male, n (%)			Female, n (%)			Overall, n (%)
	Fourth	Fifth	Intern	Fourth	Fifth	Intern	
Use of tobacco products	54 (13.8)	49 (12.5)	21 (5.4)	84 (21.4)	120 (30.6)	20 (5.1)	348 (88.8)
Use of alcohol	47 (12.0)	40 (10.2)	19 (4.8)	83 (21.2)	110 (28.1)	17 (4.3)	316 (80.6)
Human Papilloma Virus	39 (9.9)	32 (8.2)	19 (4.9)	74 (18.9)	94 (24.0)	17 (4.3)	275 (70.2)
Prior oral cancer lesion	51 (13.0)	46 (11.7)	20 (5.1)	82 (20.9)	114 (29.1)	19 (4.9)	332 (84.7)
Family history of oral cancer	5 (1.3)	5 (1.3)	0 (0.0)	10 (2.5)	9 (2.3)	0 (0.0)	29 (7.4)
Low consumption of fruits and vegetables	22 (5.6)	17 (4.3)	3 (0.8)	27 (6.9)	29 (7.4)	6 (1.5)	104 (26.5)
Poorly fitting dentures	37 (9.4)	16 (4.1)	12 (3.1)	27 (6.9)	41 (10.4)	7 (1.8)	140 (35.7)
Sun exposure (for lip cancer)	39 (9.9)	40 (10.2)	19 (4.9)	68 (17.4)	115 (29.3)	19 (4.8)	300 (76.5)
Older age	51 (13.0)	45 (11.5)	18 (4.6)	76 (19.4)	112 (28.5)	18 (4.6)	320 (81.6)
Use of spicy food	37 (9.4)	27 (6.9)	12 (3.1)	50 (12.7)	50 (12.8)	11 (2.8)	187 (47.7)
Hot beverages and foods	33 (8.4)	27 (6.9)	13 (3.3)	48 (12.2)	59 (15.1)	13 (3.3)	193 (49.2)
Poor oral hygiene	19 (4.8)	12 (3.1)	7 (1.8)	31 (7.9)	31 (7.9)	4 (1.0)	104 (26.5)

**Table 4:** Number and percentage of undergraduate dental students and interns who claimed good practice and experience with oral cancer screening and referral at AAUP

Question	Survey results						
	Male, n (%)			Female, n (%)			Overall, n (%)
	Fourth	Fifth	Intern	Fourth	Fifth	Intern	
How often do you screen your patients for signs of oral cancer?	33 (8.4)	22 (5.6)	7 (1.8)	43 (11.0)	38 (9.7)	8 (2.0)	151 (38.5)
Do you discuss the risk factors of oral cancer with your patients?	18 (4.6)	14 (3.6)	4 (1.0)	32 (8.1)	17 (4.3)	3 (0.8)	88 (22.4)
Do you use diagnostic aids to help you detect suspicious oral mucosal lesions?	45 (11.5)	27 (6.9)	6 (1.5)	60 (15.3)	61 (15.6)	8 (2.0)	207 (52.8)
Have you ever detected a suspicious oral mucosal lesion?	24 (6.1)	21 (5.3)	13 (3.3)	32 (8.2)	41 (10.5)	11 (2.8)	142 (36.2)
Have you ever referred to a specialist for a suspicious oral mucosal lesion?	28 (7.1)	18 (4.6)	16 (4.1)	31 (7.9)	33 (8.4)	12 (3.1)	138 (35.2)
Have you been instructed on how to perform an incisal or punch biopsy of a suspicious lesion in the oral cavity?	27 (6.9)	17 (4.4)	9 (2.3)	35 (8.9)	39 (9.9)	7 (1.8)	134 (34.2)
Have you been instructed to use brush biopsy?	35 (8.9)	22 (5.6)	6 (1.5)	37 (9.5)	36 (9.2)	9 (2.3)	145 (37.0)
Have you been instructed to use VizaLite?	20 (5.1)	8 (2.0)	1 (0.3)	17 (4.3)	14 (3.6)	1 (0.3)	61 (15.6)
Have you been instructed to use Toluidine blue?	28 (7.1)	8 (2.0)	3 (0.8)	31 (7.9)	22 (5.6)	5 (1.3)	97 (24.7)
Thus far in your training, about how many oral lesion biopsies have you seen?	2 (0.5)	10 (2.6)	7 (1.8)	13 (3.3)	13 (3.3)	4 (1.0)	49 (12.5)
Do you give smoking cessation advice to patients who smoke?	54 (13.8)	43 (11.0)	18 (4.6)	85 (21.7)	108 (27.5)	15 (3.8)	323 (82.4)
Do you provide any educational material for patients regarding the risks of oral cancer?	40 (10.2)	30 (7.6)	7 (1.8)	59 (15.1)	62 (15.8)	7 (1.8)	205 (52.3)

**Table 5:** Number and percentage of undergraduate dental students and interns who revealed positive attitudes toward oral cancer prevention

Question	Survey results (agree)						
	Male, n (%)			Female, n (%)			Overall, n (%)
	Fourth	Fifth	Intern	Fourth	Fifth	Intern	
I am adequately trained to examine patients for oral cancer	35 (8.9)	24 (6.1)	13 (3.3)	39 (10.0)	40 (10.2)	11 (2.8)	162 (41.3)
I am adequately trained to palpate cervical lymph nodes	51 (13.0)	42 (10.7)	18 (4.6)	79 (20.2)	97 (24.7)	17 (4.3)	304 (77.5)
I am adequately trained to refer patients high risk oral lesions to specialists	45 (11.5)	31 (7.9)	20 (5.1)	57 (14.5)	69 (17.6)	18 (4.6)	240 (61.2)
I am adequately trained to provide tobacco cessation education	40 (10.2)	39 (9.9)	17 (4.3)	72 (18.4)	81 (20.7)	18 (4.6)	267 (68.1)
I am adequately trained to provide alcohol cessation education	43 (11.0)	32 (8.2)	15 (3.8)	69 (17.6)	69 (17.6)	16 (4.1)	244 (62.3)
It is the role of the dentist to screen for oral mucosal pathology	51 (13.0)	45 (11.5)	19 (4.9)	82 (20.9)	117 (29.8)	20 (5.1)	334 (85.2)
I should provide my patients smoking cessation counseling and advice	54 (13.8)	50 (12.8)	19 (4.8)	83 (21.2)	122 (31.1)	20 (5.1)	348 (88.8)
Screening of oral mucosal soft tissues should occur at new patient appointments	57 (14.5)	49 (12.5)	19 (4.9)	80 (20.4)	118 (30.1)	20 (5.1)	343 (87.5)
Screening of oral mucosal soft tissues should occur at recall patient appointments	48 (12.2)	47 (12.0)	18 (4.6)	83 (21.2)	110 (28.1)	17 (4.3)	323 (82.4)
Do you think you need additional training in oral cancer prevention and screening?	60 (15.3)	51 (13.0)	21 (5.4)	92 (23.5)	124 (31.6)	20 (5.1)	368 (93.9)

Concerning the assessed knowledge of oral cancer and its risk factors, 66.8% of the respondents had a poor knowledge with an 11.86 (3.56) overall mean among the responding participants. About 85.5% of the responding participants had a poor practice of oral cancer prevention and early detection with an overall mean of 4.44 (2.75). However, most respondents (81.1%) had favorable attitudes toward oral cancer prevention with an overall mean of 7.48 (2.15) (Table 6).

**Table 6:** Knowledge, practice, and attitude scores of undergraduate dental students and interns at AAUP about oral cancer prevention (n = 392)

Variables	N	%	Mean (SD)
Knowledge			11.86 (3.56)
Poor (0–13.2)	262	66.8	
Good (> 13.2)	130	33.2	
Practice			4.44 (2.75)
Poor (0–7.2)	335	85.5	
Good (> 7.2)	57	14.5	
Attitude			7.48 (2.15)
Unfavorable (0–6)	74	18.9	
Favorable (> 6)	318	81.1	

Regarding if the participant’s age, sex, or education level had a significant effect on the level of knowledge, practice, and attitudes toward oral cancer prevention, an independent t-test and ANOVA showed that sex was significantly associated with oral cancer practice in the College of Dentistry at AAUP (*P* < 0.05). Male responders (4.94 ± 2.86) had better practice scores compared to females (4.16 ± 2.65). Education level was also significantly related to knowledge, practice, and attitudes toward oral cancer prevention in the College of Dentistry at AAUP (*P* < 0.05). Interns had significantly better knowledge (13.68 ± 2.43) and attitude scores (8.68 ± 1.68) toward oral cancer prevention compared to undergraduate dental students; however, fourth-year dental students had significantly better practice scores (4.93 ± 2.90) when compared to fifth-year students and interns (Table 7).

**Table 7:** Knowledge, practice, and attitude of oral cancer prevention among undergraduate dental students and interns at AAUP by age, sex, and education level (n = 392)

Variable	N (%)	Knowledge		Practice		Attitude	
		Mean (SD)	*P* value	Mean (SD)	*P* value	Mean (SD)	*P* value
Age			0.527*		0.313*		0.112*
≤ 30	369 (94.1)	11.90 (3.46)		4.40 (2.77)		7.44 (2.13)	
> 30	23 (5.9)	11.22 (5.04)		5.00 (2.43)		8.17 (2.44)	
Gender			0.397*		0.007*		0.212*
Male	140 (35.7)	11.66 (3.57)		4.94 (2.86)		7.66 (2.19)	
Female	252 (64.3)	11.98 (3.56)		4.16 (2.65)		7.38 (2.13)	
Education level			0.000^¶^		0.004^¶^		0.001^¶^
4th-year	168 (42.8)	10.97 (3.73)		4.93 (2.90)		7.26 (2.25)	
5th-year	183 (46.7)	12.27 (3.40)		3.96 (2.67)		7.42 (2.08)	
Intern	41 (10.5)	13.68 (2.43)		4.56 (2.03)		8.68 (1.68)	

*SD* standard deviation

^*^t-test of significance

^¶^ANOVA test of significance

Regarding the perceived barriers to oral cancer screening among undergraduate dental students and interns at AAUP, more than half of the respondents (57.2%) believed that insufficient training was a barrier. Other claimed barriers included lack of time (19.3%), lack of confidence (12.9%), and lack of effectiveness (10.6%; Table 8).

**Table 8:** Perceived barriers to oral cancer screening among undergraduate dental students and interns at AAUP

Barrier	Fourth (%)	Fifth (%)	Intern (%)	Overall (%)
I do not have sufficient time	36 (10.3)	20 (5.8)	11 (3.2)	67 (19.3)
I am not trained enough to do so	73 (21.0)	108 (31.0)	18 (5.2)	199 (57.2)
I do not have confidence to do so	28 (8.0)	16 (4.6)	1 (0.3)	45 (12.9)
I think this would not be effective	23 (6.6)	13 (3.7)	1 (0.3)	37 (10.6)

*AAUP* Arab American University

## Discussion

This cross-sectional study sought to assess the knowledge, practices, and attitudes toward oral cancer prevention and early detection among students and interns in the College of Dentistry at AAUP; and to investigate the factors that influence their practices of oral cancer screening or prevention.

There was an 68.8% response rate, which is higher compared to other analogous studies from Saudi Arabia (56.4%, 54.2%) [27, 32].

The current study revealed that the level of oral cancer knowledge among most of the surveyed participants is regarded as poor. This result is comparable to the results of previous studies conducted in Saudi Arabia [31, 32], Kuwait [30] and United Arab Emirates [34], but it is in contrast to those of other studies in Saudi Arabia [18, 28] and India [25] that revealed good to excellent knowledge of oral cancer and its associated risk factors among dental students and interns.

Regarding the level of the practice of oral cancer prevention and early detection, the present survey demonstrated a poor level of practice among the surveyed participants. This is in accordance with the results of a recent cross-sectional study in Saudi Arabia [32]; however, it is in contrary to the results of studies in India [25] and Brazil [24] that demonstrated good practices of oral cancer prevention and screening among undergraduate dental students. Might be the poor level of oral cancer knowledge in the current study justifies the poor level of practice among the responding participants.

In addition, the present study demonstrated that the attitude toward oral cancer prevention among the participants was favorable. This agrees with the results of other studies in Saudi Arabia [31], India [25] and Brazil [24], but contrasts with those of a recent cross-sectional study conducted in Saudi Arabia [32].

The current study concluded that the level of education positively affected the knowledge and attitude scores; since the interns in the College of Dentistry at AAUP had significantly better knowledge and attitude scores toward oral cancer prevention compared to the undergraduate dental students. This finding is in accordance with that demonstrated by Shubayr et al. [32], but it is in contrast to the results of a study in Turkey [19] that demonstrated no significant association between the year of study in the dental college and the level of knowledge of oral cancer risk factors.

Regarding the practice of oral cancer prevention and early detection, why fourth-year dental students had significantly better practice scores compared to fifth-year students and interns might be explained by their application of freshly educated topics since they start to take these topics at fourth-year level.

Concerning the perceived barriers to oral cancer prevention and screening among the undergraduate dental students and interns at AAUP, lack of training was the most prevalent, followed by lack of time, lack of confidence, and lack of effectiveness. These findings are in accordance with the results of a study [26] undertaken in Australia that reported that lack of training, confidence, time, and financial incentives were seen as barriers to performing oral mucosal screening to at least some degree by the responding participants. The lack of training could be managed by enhancing the dental undergraduate curricula and by making continuous educational programs and training on oral cancer prevention and early detection. In the United States, the CODA (Commission on Dental Accreditation) specifies that all USA dental students should be experienced in screening for head and neck cancer and in identification of its risk factors [35]. It is worth noting that the studies published before the recent modifications of the CODA academic standard had revealed that American dental students and dentists considered themselves undertrained in oral cancer screening and detection [20, 21].

Undergraduate dental students in AAUP are sensitized to the subject of oral cancer starting from the third year of curriculum. During this year, the students in oral pathology course are exposed to risk factors and carcinogenesis of oral cancer and potentially malignant oral disorders. This course is given in the form of lectures, structured interactive sessions, and evaluation of histopathological slides. By the fourth and fifth years, the students in oral medicine courses get the theoretical basics of diagnosis and treatment of malignant and potentially malignant oral lesions in the form of lectures, structured interactive sessions, and seminars. These sessions are guided by oral medicine and oral surgery specialists. Clinically, the students in the fifth year have an oral medicine clinic where potentially malignant oral lesions and oral cancer cases are referred to and the students practice examining, diagnosing, and treating like these cases.

It appears that the present study is the first in Palestine to assess the knowledge, practice, and attitudes toward oral cancer prevention and early detection among future and current dentists.

This study gains its importance from being a cross-sectional, low-cost, and prompt method that provides useful information on the adequacy of the dental curriculum of oral cancer prevention in this dental school, and also gives insight into the need for continuous education programs to train students and practitioners in oral cancer prevention theoretically and practically.

However, caution should be taken when interpreting the results of the current study due to some methodological limitations. The study was a questionnaire-based survey and all data were self-reported and subjective; therefore, the responses may not appropriately reproduce the real levels of knowledge and attitudes. In addition, this study included only dental students and interns in the College of Dentistry at AAUP; this may limit the generalizability of the findings to all students and dentists practicing in Palestine. Finally, the relatively low response rate (68.8%) that could introduce a nonresponse bias should be taken into consideration. However, this study shed the light on the necessity of enhancing the educational undergraduate curricula and on conducting continuous training activities for dental students and dentists about oral cancer prevention and early detection both theoretically and practically. Numerous oral cancer screening cases should be part of dental students’ clinical requirements. Dental schools should be the leader in heading like these training programs for oral health providers; therefore, oral cancer cases will be early detected leading to an increase in oral cancer survival rates and a decrease of morbidity rates [36, 37].

## Conclusions

According to the findings of this survey, it was concluded that dental students and interns at Arab American University in Palestine appeared to have a good attitude and a good motivation toward oral cancer prevention and early detection. However, they lacked the adequate knowledge and training. Interns had higher knowledge and attitude scores toward oral cancer prevention compared to the undergraduate dental students. More efforts and further research are needed to fill the gap in oral cancer knowledge and training by enhancing the undergraduate curricula and by organizing periodic, continuous training activities for dental students and dentists with regard to oral cancer prevention and early detection at the AAUP College of Dentistry.

## Data Availability

The data that support the findings of this study are submitted with the manuscript.

## Abbreviations

AAUP: Arab American University
MENA: Middle East and North Africa
ANOVA: One way analysis of variance
CODA: Commission on dental accreditation

## References

[1] Sung H, Ferlay J, Siegel RL, Laversanne M, Soerjomataram I, Jemal A, Bray F (2021). Global Cancer Statistics 2020: GLOBOCAN estimates of incidence and mortality worldwide for 36 cancers in 185 countries. CA Cancer J Clin.

[2] Warnakulasuriya S (2009). Global epidemiology of oral and oropharyngeal cancer. Oral Oncol.

[3] Kujan O, Farah CS, Johnson NW (2017). Oral and oropharyngeal cancer in the Middle East and North Africa: incidence, mortality, trends, and gaps in public databases as presented to the Global Oral Cancer Forum. Transl Res Oral Oncol..

[4] Ferlay J, Soerjomataram I, Ervik M, et al. GLOBOCAN 2012 v1.0, cancer incidence and mortality worldwide: IARC CANCER Base No. 11. Lyon: International Agency for Research on Cancer; 2013. http://globocan.iarc.fr.

[5] El-Naggar AK, Chan JKC, Grandis JR, Takata T, Slootweg PJ, editors. WHO classification of head and neck tumours. 4th ed.Lyon: International Agency for Research on Cancer; 2017. p. 347. (World Health Organization classification of tumors).

[6] Scully C, Kirby J (2014). Statement on mouth cancer diagnosis and prevention. Br Dent J.

[7] Chi AC, Day TA, Neville BW (2015). Oral cavity and oropharyngeal squamous cell carcinoma—an update. CA Cancer J Clin.

[8] Al-Rawi NH, Talabani NG (2008). Squamous cell carcinoma of the oral cavity: a case series analysis of clinical presentation and histological grading of 1,425 cases from Iraq. Clin Oral Investig.

[9] Subhashraj K, Orafi M, Nair KV, El-Gehani R, Elarbi M (2009). Primary malignant tumors of orofacial region at Benghazi, Libya: a 17 years review. Cancer Epidemiol.

[10] Abdul-Hamid G, Saeed NM, Al-Kahiry W, Shukry S (2010). Pattern of head and neck cancer in Yemen. Gulf J Oncolog.

[11] Vaccarella S, Bruni L, Seoud M (2013). Burden of human papillomavirus infections and related diseases in the extended Middle East and North Africa region. Vaccine.

[12] World Health Organization (2009). Regional office for the Eastern Mediterranean. Towards a strategy for cancer control in the Eastern Mediterranean region.

[13] GBD 2017 Disease and Injury Incidence and Prevalence Collaborators (2018). Global, regional, and national incidence, prevalence, and years lived with disability for 354 diseases and injuries for 195 countries and territories, 1990–2017: a systematic analysis for the Global Burden of Disease Study 2017. Lancet.

[14] Ren ZH, Hu CY, He HR, Li YJ, Lyu J (2020). Global and regional burdens of oral cancer from 1990 to 2017: results from the global burden of disease study. Cancer Commun.

[15] Silverman S, Kerr AR, Epstein JB (2010). Oral and pharyngeal cancer control and early detection. J Cancer Educ.

[16] Sankaranarayanan R, Ramadas K, Amarasinghe H, Subramanian S, Johnson N. Oral cancer: prevention, early detection, and treatment. In: Gelband H, Jha P, Sankaranarayanan R, Horton S, editors. Cancer: Disease Control Priorities, Third Edition (Volume 3). Washington (DC): The International Bank for Reconstruction and Development/The World Bank; 2015. Available from: http://www.ncbi.nlm.nih.gov/books/NBK343649/]. 26913350

[17] Grafton-Clarke C, Chen KW, Wilcock J (2019). Diagnosis and referral delays in primary care for oral squamous cell cancer: a systematic review. Br J Gen Pract.

[18] Kujan O, Alzoghaibi I, Azzeghaiby S, Altamimi MA, Tarakji B, Hanouneh S, Idress M, Alenzi FQ, Iqbal M, Taifour S (2014). Knowledge and attitudes of Saudi dental undergraduates on oral cancer. J Cancer Educ.

[19] Keser G, Pekiner FN (2019). Assessing oral cancer awareness among dental students. J Cancer Educ.

[20] Burzynski NJ, Rankin KV, Silverman S, Scheetz JP, Jones DL (2002). Graduating dental students' perceptions of oral cancer education: results of an exit survey of seven dental schools. J Cancer Educ.

[21] Cannick GF, Horowitz AM, Drury TF, Reed SG, Day TA (2005). Assessing oral cancer knowledge among dental students in South Carolina. J Am Dent Assoc..

[22] López-Jornet P, Camacho-Alonso F, Molina-Miñano F (2010). Knowledge and attitudes about oral cancer among dentists in Spain. J Eval Clin Pract.

[23] Decuseara G, MacCarthy D, Menezes G (2011). Oral cancer: knowledge, practices and opinions of dentists in Ireland. J Ir Dent Assoc.

[24] Soares TR, Carvalho ME, Pinto LS (2014). Oral cancer knowledge and awareness among dental students. Braz J Oral Sci.

[25] Fotedar S, Bhardwaj V, Manchanda K, Fotedar V, Sarkar AD, Sood N (2015). Knowledge, attitude and practices about oral cancers among dental students in H.P Government Dental College, Shimla-Himachal Pradesh. South Asian J Cancer..

[26] Allen K, Farah CS (2015). Screening and referral of oral mucosal pathology: a check-up of Australian dentists. Aust Dent J.

[27] Khan SQ, Farooqi FA, Moheet IA, Rejaie ASA (2016). Attitude and experiences of undergraduate dental students and interns towards research. Saudi J Med Med Sci.

[28] Jafer M, Crutzen R, Jafer A, van den Borne B (2018). What do dental college clinicians know about oral cancer and its risk factors? An assessment among final year students, interns and faculty members in saudi arabia. J Clin Exp Dent.

[29] Ahmed NHM, Naidoo S (2019). Oral cancer knowledge, attitudes, and practices among dentists in Khartoum State. Sudan J Cancer Educ.

[30] Nazar H, Shyama M, Ariga J, El-Salhy M, Soparkar P, Alsumait A (2019). Oral cancer knowledge, attitudes and practices among primary oral health care dentists in Kuwait. Asian Pac J Cancer Prev..

[31] Alsaud B (2019). Knowledge, attitudes, and practices of dental undergraduates and practitioners regarding oral cancer in Jeddah. Saudi Arabia EC Dent Sci.

[32] Shubayr MA, Bokhari AM, Essa AA, Nammazi AM, Al Agili DE (2021). Knowledge, attitudes, and practices of oral cancer prevention among students, interns, and faculty members at the college of dentistry of Jazan University. BMC Oral Health.

[33] Eysenbach G. Improving the quality of Web surveys: the Checklist for Reporting Results of Internet E-Surveys (CHERRIES) [published correction appears in doi:10.2196/jmir.2042]. J Med Internet Res. 2004;6(3):e34. 10.2196/jmir.6.3.e34 10.2196/jmir.6.3.e34 PMC1550605 15471760

[34] Gaballah K, Faden A, Fakih FJ, Alsaadi AY, Noshi NF, Kujan O (2021). Diagnostic accuracy of oral cancer and suspicious malignant mucosal changes among future dentists. Healthcare.

[35] American Dental Association (2013). Accreditation standards for dental education programs.

[36] Ford P, Farah C (2013). Early detection and diagnosis of oral cancer: strategies for improvement. J Cancer Policy.

[37] Mariño R, Haresaku S, McGrath R, Bailey D, Mccullough M, Musolino R, Kim B, Chinnassamy A, Morgan M (2017). Oral cancer screening practices of oral health professionals in Australia. BMC Oral Health.

